# Combined Ultrasound and Photoacoustic Image Guidance of Spinal Pedicle Cannulation Demonstrated With Intact *Ex Vivo* Specimens

**DOI:** 10.1109/TBME.2020.3046370

**Published:** 2021-07-19

**Authors:** Eduardo A. Gonzalez, Amit Jain, Muyinatu A. Lediju Bell

**Affiliations:** Department of Biomedical Engineering, Johns Hopkins University, Baltimore, MD 21218 USA; Department of Orthopaedic Surgery, Johns Hopkins University; Department of Electrical and Computer Engineering, the Department of Computer Science, and the Department of Biomedical Engineering, Johns Hopkins University

**Keywords:** Bone segmentation, coherence imaging, landmark registration, photoacoustic imaging, spinal fusion

## Abstract

**Objective:**

Spinal fusion surgeries require accurate placement of pedicle screws in anatomic corridors without breaching bone boundaries. We are developing a combined ultrasound and photoacoustic image guidance system to avoid pedicle screw misplacement and accidental bone breaches, which can lead to nerve damage.

**Methods:**

Pedicle cannulation was performed on a human cadaver, with co-registered photoacoustic and ultrasound images acquired at various time points during the procedure. Bony landmarks obtained from coherence-based ultrasound images of lumbar vertebrae were registered to post-operative CT images. Registration methods were additionally tested on an *ex vivo* caprine vertebra.

**Results:**

Locally weighted short-lag spatial coherence (LW-SLSC) ultrasound imaging enhanced the visualization of bony structures with generalized contrast-to-noise ratios (gCNRs) of 0.99 and 0.98–1.00 in the caprine and human vertebrae, respectively. Short-lag spatial coherence (SLSC) and amplitude-based delay-and-sum (DAS) ultrasound imaging generally produced lower gCNRs of 0.98 and 0.84, respectively, in the caprine vertebra and 0.84–0.93 and 0.34–0.99, respectively, in the human vertebrae. The mean ± standard deviation of the area of −6 dB contours created from DAS photoacoustic images acquired with an optical fiber inserted in prepared pedicle holes (i.e., fiber surrounded by cancellous bone) and holes created after intentional breaches (i.e., fiber exposed to cortical bone) was 10.06±5.22 mm^2^ and 2.47±0.96 mm^2^, respectively (p < 0.01).

**Conclusions:**

Coherence-based LW-SLSC and SLSC beamforming improved visualization of bony anatomical landmarks for ultrasound-to-CT registration, while amplitude-based DAS beamforming successfully distinguished photoacoustic signals within the pedicle from less desirable signals characteristic of impending bone breaches.

**Significance:**

These results are promising to improve visual registration of ultrasound and photoacoustic images with CT images, as well as to assist surgeons with identifying and avoiding impending bone breaches during pedicle cannulation in spinal fusion surgeries.

## I. Introduction

Spinal instability can be caused by degenerative disorders, trauma, and primary or metastatic cancer [1]. These abnormalities are commonly treated with spinal fusion surgeries, which help to alleviate pain and recover neurological functionality. Contemporary posterior spinal fusion surgeries involve drilling holes into the pedicles of vertebrae, inserting pedicle screws, and attaching each screw to a metal rod with the goal of stabilizing the spine to allow for bony fusion to occur. When cannulating pedicles, it is critical to ensure the correct trajectory during drilling in order to avoid accidental bone breaches and screw misplacement. In particular, pedicle screw misplacement occurs in approximately 14–39.8% of procedures [2]–[5], which compromises neighboring structures such as nerves (and in some cases, spinal cord), erodes long-term biomechanical stability, [6], [7], and causes adjacent degeneration [8], [9].

Computer assisted spinal surgery methods are becoming increasingly prevalent, as they improve the accuracy of pedicle screw placement and patient outcomes in comparison to the conventional free-hand screw fixation [10]. Currently, these methods utilize computed tomography (CT) [11], 2D or 3D fluoroscopy navigation [12], [13], and robotic assistance [14] to provide intraoperative information, which can be registered to preoperative CT images. The surgical tool and anatomical landmarks are then identified in the registered images to help surgeons localize the pedicle anatomic corridor location. However, limitations of these methods include exposure to ionizing radiation, the requirement to insert reference and intraoperative markers, and relatively prolonged surgery times.

Ultrasound imaging is a safer alternative to potentially provide real-time intraoperative information for pre-operative CT image registration [15]. However, limitations with ultrasound imaging for pedicle screw guidance include sound attenuation in the presence of bone, poor signal-to-noise ratios (SNRs), and the presence of clutter and speckle noise. Conventional ultrasound imaging has limited ability to detect deep-lying features beneath bone tissue due to sound attenuation and sound speed differences, requiring the use of several ultrasound slices as redundant information for registration (i.e., 3D ultrasound imaging) [16]–[18]. The feasibility of multiple slice registration is accomplished with additional tracking devices and custom hardware [19].

To overcome limitations with ultrasound imaging, photoacoustic imaging [20] has been proposed as a guidance method for pedicle screw insertion [21]. The proposed technique consists of delivering laser light to generate an acoustic pressure response. The acoustic pressure is then received by an ultrasound probe, and beamforming is applied to create a photoacoustic image. Applications of photoacoustic imaging to surgical guidance include visualization of tool tips such as a neurosurgical drill tip [22], a needle tip [23]–[26], or a cardiac catheter tip [27], visualization of underlying structures such as blood vessels [28], and photoacoustic-based guidance during a range of surgeries, such as fetal surgeries [29], endonasal surgeries, [30]–[32], hysterectomy procedures [33], [34], and prostate surgeries [35]. The incorporation of robotics with teleoperated surgery [33] and robotic visual servoing [36], [37] has also been demonstrated. Applications related to the spine include stem cell injection guidance [38] and discrimination of cortical bone from cancellous bone to identify optimal insertion points prior to initiating pedicle screw placement [21]. Despite these remarkable advances, no previous studies investigate the accuracy of photoacoustic signal visualization and localization within the pedicle of a vertebra.

This paper investigates two hypotheses. First, based on previous studies to visualize photoacoustic signals from the surface of human vertebrae [21], we hypothesize that similar visibility can be achieved beneath the bony structure in a more realistic setup and closer to the surgical environment of a spinal fusion surgery. Second, we hypothesize that improvements to 2D ultrasound imaging would reduce the computational burden associated with requiring 3D ultrasound images to complete the segmentation task for ultrasound-to-CT registration. To address the poor 2D ultrasound segmentation that otherwise compromises the performance of ultrasound-to-CT registration, we propose a novel coherence-based beamforming technique named locally weighted short-lag spatial coherence (LW-SLSC) beamforming. LW-SLSC beamforming is a regularized version of short-lag spatial coherence (SLSC) beamforming [39], designed to minimize the trade off between contrast and spatial resolution. Therefore, LW-SLSC has the potential to enhance the vertebral boundaries adjacent to soft tissue when compared to conventional delay-and-sum (DAS) beamforming, as previously demonstrated in an *ex vivo* caprine vertebra [40].

Our hypotheses were tested with *ex vivo* caprine and human vertebrae. First, the segmentation enhancement achieved with LW-SLSC beamforming was compared to that obtained from SLSC beamforming and conventional DAS beamforming in an *ex vivo* caprine vertebra. Then, we demonstrated the visualization of photoacoustic signals originating from inside the lumbar vertebrae located inside a human cadaver during pedicle hole creation, using the same methods implemented during spinal fusion surgeries. Validation of the photoacoustic signal locations was based on manual registration of post-operative CT volumes to co-registered ultrasound and photoacoustic images. This registration relied on identified landmarks within segmented ultrasound images that were enhanced with LW-SLSC beamforming. Finally, we successfully differentiated photoacoustic signals originating from cancellous and cortical bone inside the human cadaver by measuring the areas of −6 dB contours of DAS photoacoustic images.

This paper is organized as follows. Section II details our acquisition, beamforming, segmentation, and registration methods. Section III presents our experimental results. Section IV discusses insights from the experimental results. Section V summarizes our conclusions.

## II. Methods

### A. Coherence-Based Beamforming Methods

#### 1) Short-Lag Spatial Coherence:

Unlike the conventional amplitude-based DAS beamformer, SLSC beamforming [39] displays the similarity of received signals in the aperture domain, as a function of element separation *m*. A received time-delayed sample is represented as *s*_*i*_(*n*), where *i* is the channel index and *n* is the depth index in a zero-mean radio frequency signal *s*_*i*_. First, the coherence function *R*(*m*) is calculated using an axial kernel as follows:

(1) $$\hat{R}(m)=\frac{1}{N-m}\sum_{i=1}^{N-m}\frac{\sum_{n=n_{1}}^{n_{2}}s_{i}(n)s_{i+m}(n)}{\sqrt{\sum_{n=n_{1}}^{n_{2}}s_{i}^{2}(n)\sum_{n=n_{1}}^{n_{2}}s_{i+m}^{2}(n)}},$$

where *N* is the the number of elements in the aperture, and *n*_1_ and *n*_2_ are the limits of the axial kernel *k* in units of sample number. Then, an SLSC image is generated as the integral of the spatial coherence function over the first *M* lags:

(2) $$\mathrm{SLSC}(M)=\int_{1}^{M}\hat{R}(m)dm\approx \sum_{m=1}^{M}\hat{R}(m).$$

#### 2) Locally Weighted Short-Lag Spatial Coherence:

Enhancement of bone boundaries can be achieved by implementing a regularized version of the SLSC beamformer [40]. Instead of averaging the cumulative sum up to a lag value *M* (out of a preselected total of *N*_*L*_ lags, where M < *N*_*L*_), LW-SLSC beamforming computes the weighted coefficients for *N*_*L*_ lags by minimizing the total variation (TV) of the weighted sum within a moving kernel ${\hat{R}}_{i}\in {\mathbb{R}}^{k_{z}\times k_{x}\times N_{L}}$ obtained from the correlation matrix $\hat{R}\in {\mathbb{R}}^{N_{z}\times N_{x}\times N_{L}}$. In order to preserve the high resolution information available at higher lags (i.e., M > 15), this adaptive solution was regularized using the L2-norm with a gradient operator. Then, the TV minimization was defined as:

(3) $$\;{\hat{w}}_{i}={\mathrm{argmin}}_{w_{i}}\left\{\text{TV}\left(f\left(w_{i},{\hat{R}}_{i}\right)\right)+\alpha^{2}{\left\|\nabla w_{i}\right\|}_{2}^{2}\right\}\;f\left(w_{i},{\hat{R}}_{i}\right)=\sum_{m=1}^{N_{L}}{\hat{R}}_{i}[m]\cdot w_{i}[m]\;\;\text{Subject}\;\text{to :}\;{\left\|w_{i}\right\|}_{1}=1\;\;0\leq w_{i}\leq 1$$

where TV is the 2D total variation with the L2-norm applied to the cost function *f*, ${\hat{R}}_{i}$ is the kernel *i* of the correlation matrix $\hat{R}$, and $w_{i}\in {\mathbb{R}}^{1\times N_{L}}$ is the optimized weight vector for the calculated summed lags of ${\hat{R}}_{i}$. The weighted sum kernels ${\hat{w}}_{i}{\hat{R}}_{i}$ were stacked into multiple layers and positioned relative to the center of each ${\hat{R}}_{i}$. The LW-SLSC image was the median of the stacked kernels ${\hat{w}}_{i}{\hat{R}}_{i}$. The main advantage of LW-SLSC relies on the adaptive selection of lower lags in kernels surrounding isoechoic regions, which enhances contrast, and higher lags otherwise, which enhances resolution. The selective combination of higher and lower lags is known to reduce the noise commonly observed in SLSC images created with higher lags [41].

The original formulation in (3) can be simplified using the framework of Barbero *et al.* [42] for Total Variance alternatives. However, these simplifications only hold when computing TV with the L2-norm. The gradient operator ∇ (i.e., 1D TV operator) used in the penalty term is simplified to:

(4) $$D=\left(\begin{array}{cccccc} -1 & 1 & & & & \\ & -1 & 1 & & & \\ & & \ddots & \ddots & & \\ & & & & -1 & 1 \end{array}\right)\;{\text{TV}}_{1D}\left(w_{i}\right)={\left\|Dw_{i}\right\|}_{p}\;D\in {\mathbb{R}}^{\left(N_{L}-1\right)\times N_{L}}$$

Similarly, the two dimensional TV operator used in the fidelity term is reduced to:

(5) $$B=\left(\begin{array}{cccccccccc} -1 & 1 & & & & & & & & \\ & \ddots & \ddots & & & & & & & \\ & & -1 & 1 & & & & & & \\ & & & & \ddots & \ddots & & & & \\ & & & & & & -1 & 1 & & \\ & & & & & & & \ddots & \ddots & \\ & & & & & & & & -1 & 1\\ -1 & & & & 1 & & & & & \\ & & \ddots & & & & \ddots & & & \\ & & & & & -1 & & & & 1 \end{array}\right)\;{\text{TV}}_{2D}(X)\approx \|BX{\|}_{p},\;B\in {\mathbb{R}}^{\left(2k_{z}k_{x}-k_{z}-k_{x}\right)\times \left(k_{z}k_{x}\right)}$$

Reshaping $\hat{R}$ into the form ${\hat{R}}_{i}\in {\mathbb{R}}^{k_{z}k_{x}\times N_{L}}$ and using (4) and (5) in (3), results in the following expression:

(6) $$\hat{w}={\mathrm{argmin}}_{w}\left\{{\left\|B{\hat{R}}_{i}w\right\|}_{2}^{2}+\alpha^{2}\|Dw{\|}_{2}^{2}\right\}$$

(7) $$\;={\mathrm{argmin}}_{w}\left\{w^{T}Hw\right\},\;H={\left(B{\hat{R}}_{i}\right)}^{T}B{\hat{R}}_{i}+\alpha^{2}D^{T}D$$

The reduction presented in (7) has several advantages over (3). First, by assuming the kernel size is constant during the LW-SLSC computation, the term *α*^2^*D*^*T*^ *D* is independent from the kernel *R*_*i*_ and thus can be computed only once. Second, matrix operations in ${\left(B{\hat{R}}_{i}\right)}^{T}B{\hat{R}}_{i}$ can be parallelized using built-in libraries for computational speed up, where the matrix *B* is pre-computed. Finally, the Hessian *H* allows quadratic programming using Newton step optimizers instead of the conventional gradient descent, featuring faster convergence rates. In this study, the primal-dual interior point method [43] is used for estimating the solution of (7).

### B. Segmentation of an Ex Vivo Caprine Vertebra

**Fig. 1.**
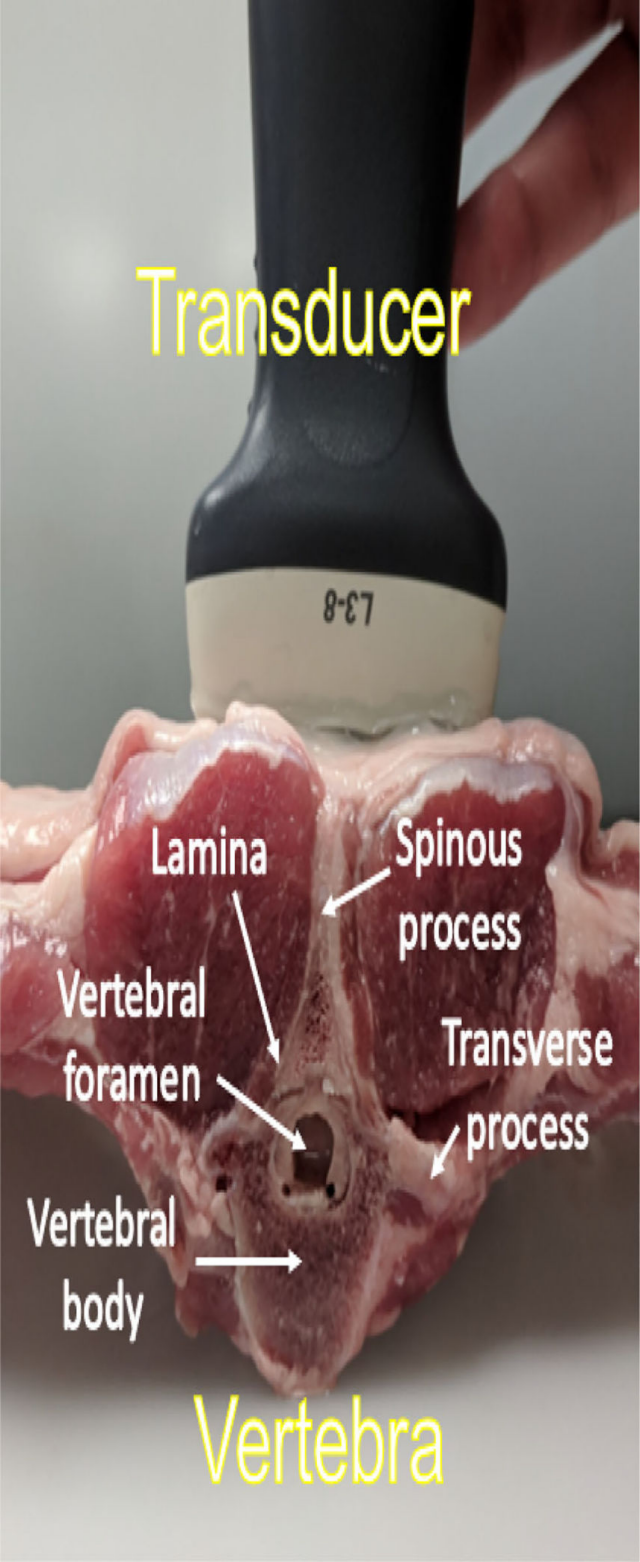
Acquisition setup for evaluating bony structure enhancement with an *ex vivo* caprine vertebra.

The segmentation accuracy of bony structures achieved with DAS, SLSC and LW-SLSC were tested on an *ex vivo* caprine thoracic vertebra (with surrounding tissue intact). This vertebra was imaged with a L3–8 linear array ultrasound probe connected to an Alpinion E-CUBE 12R ultrasound system (Alpinion, Seoul, South Korea), as shown in Fig. 1. The linear array had 128 elements, 0.3 mm pitch and 0.06 mm kerf. Raw ultrasound data were acquired with a center frequency of 4 MHz, an image depth of 40 mm, and a focus located at 30 mm depth. To compare with conventional techniques used for image guidance during spinal fusion surgeries, CT acquisitions were performed using a Siemens ARCADIS Orbic 3D C-Arm with 190 raw projections, generating a 6 cm^3^ volume of 0.12 mm^3^ voxel resolution.

SLSC images were computed with a *M* value of 9 and an axial kernel of 2*λ*, where *λ* is the wavelength of the transmit frequency. LW-SLSC images were computed with a 1.20 mm (lateral) × 1.92 mm (axial) kernel, 50% overlap, *N*_*L*_ = 28, and a regularization factor *α* = 0.12.

Bone boundaries from DAS, SLSC, and LW-SLSC images were computed by applying a binary threshold of 50% of the maximum pixel amplitude and selecting the closest contour to the vertebral foramen. These boundaries were registered with manually selected horizontal slices from volumetric 3D CT data. The registration used Mattes Mutual Information as the similarity metric [44], with One Plus One step evolutionary as the heuristic optimizer [45].

### C. Vertebral Imaging of a Human Cadaver

#### 1) Specimen and Surgery Details:

An adult male human cadaver was placed in prone position and dissection was carried along the cranio-caudal axis with the aid of a Cobb elevator to reveal the spinous process, lamina, and facet joints at each level from L1 to S1. The specimen had no reports of spine pathologies, malformations, or previous spinal surgeries, which was also confirmed with pre-operative CT imaging. The pedicles were cannulated bilaterally from L2 through L4 along anatomic trajectories using a standard free hand technique with a pedicle probe. Intentional medial and lateral breaches were made in some of the pedicle cannulation attempts. The total depth of the pedicle tracts from the bone surface ranged from 14 mm to 25 mm, as measured with the ruler on pedicle probe.

#### 2) Data Acquisition:

**Fig. 2.**
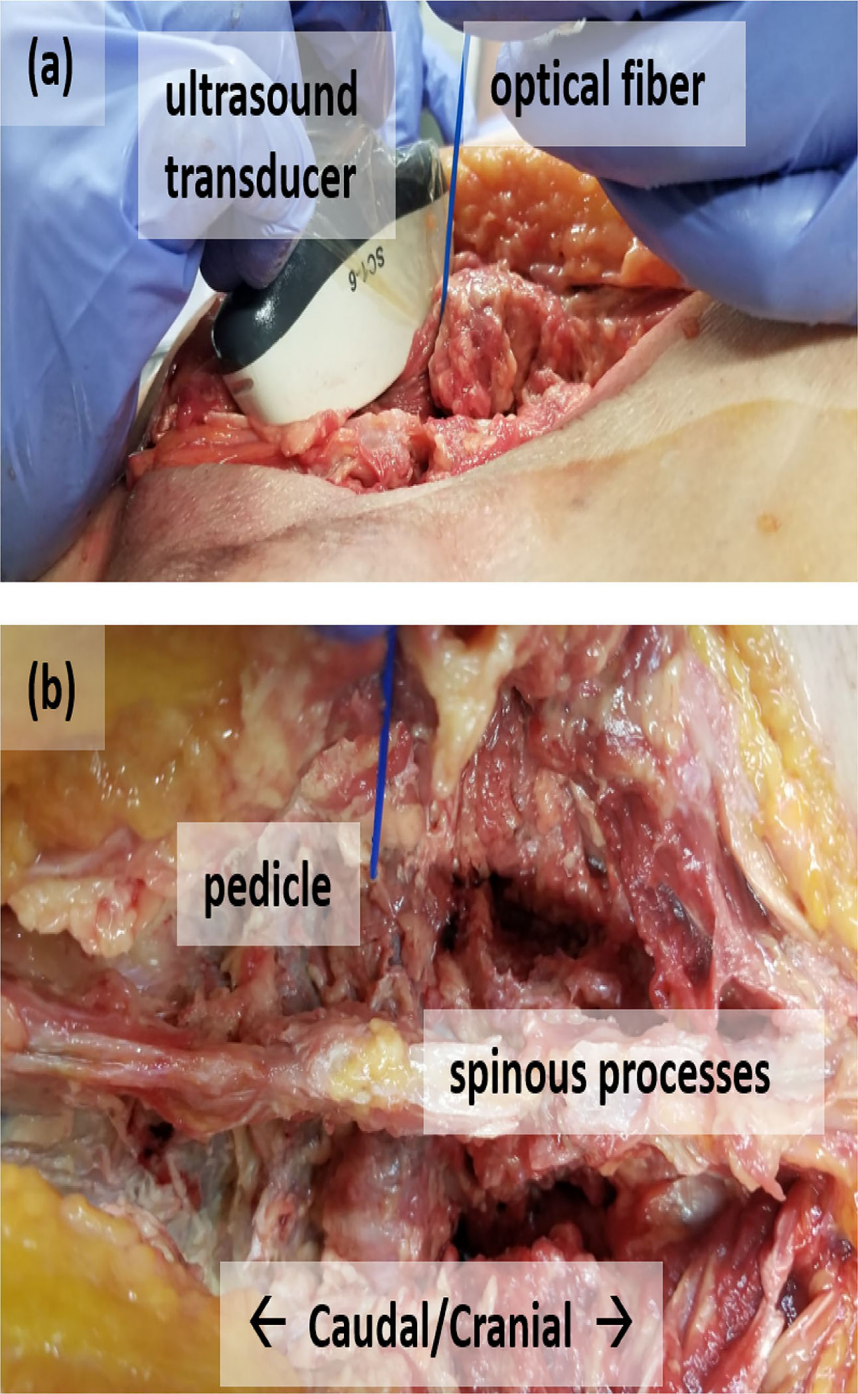
Setup to acquire ultrasound and photoacoustic data from the lumbar region of an *ex vivo* human cadaver. (a) Insertion of the optical fiber into the pedicle hole while the ultrasound transducer is placed across several lumbar laminae. (b) Posterior view of the lumbar vertebrae when the optical fiber is inserted into the pre-drilled pedicle hole.

Fig. 2 shows the acquisition setup for ultrasound and photoacoustic data from the human lumbar vertebrae. A 1-mm diameter optical fiber was inserted to touch the bottom of the pedicle hole. The optical fiber was used to transmit 750 nm wavelength laser light from a Phocus Mobile laser (Opotek Inc., Carlsbad, CA, USA) with an energy of 13.4 mJ at the fiber tip. Photoacoustic signals were received by a SC1–6 convex array ultrasound probe connected to an Alpinion E-CUBE 12R ultrasound system. The probe was positioned in an oblique axis across several lumbar laminae. Enhanced real-time visualization of photoacoustic signals was achieved with GPU implementation of SLSC [37], [46], [47] for a convex array. This photoacoustic beamforming method was chosen because it was the best real-time imaging option available to assist the surgeon with fiber tip localization during the surgery.

#### 3) Ultrasound and Photoacoustic Imaging:

Ultrasound and photoacoustic radiofrequency data were acquired up to a depth of 70 mm, with a focal depth of 25 mm for the ultrasound data. No frame averaging was applied in order to avoid the blurring artifacts that would hinder the performance of ultrasound-to-CT registration. SLSC ultrasound images were computed with *M* = 5 and 1*λ* axial kernel length, whereas LW-SLSC ultrasound images were computed with *N*_*L*_ = 15, a 2.0 mm (lateral) × 3.1 mm (axial) kernel, 60% overlap, and *α* = 1. Similarly, SLSC photoacoustic images were computed with *M* = 15 and 1*λ* axial kernel length, whereas LW-SLSC photoacoustic images were computed with *N*_*L*_ = 25, a 2.0 mm (lateral) × 3.1 (axial) kernel, 60% overlap, and *α* = 1.

#### 4) Ultrasound and Photoacoustic Segmentation:

The segmentation of bony structures and their respective centers of mass were measured from DAS, SLSC, and LW-SLSC ultrasound images, whereas the segmentation of the tip of the optical fiber and its respective center of mass was measured from DAS, SLSC, and LW-SLSC photoacoustic images. Note that the fiber tip was in contact with bone during each image acquisition, thus the fiber tip segmentation was considered to be representative of a bony landmark within the created hole. To achieve the ultrasound and photoacoustic segmentations, binary masks were computed with 30% maximum pixel amplitude threshold. Then, the removal of isolated pixels was achieved with morphological opening with a structuring element size of 0.38 mm × 0.38 mm, whereas small holes in the bony masks were filled with morphological closing with a structuring element size of 0.63 mm × 0.63 mm. Ultrasound and photoacoustic images were filtered with the computed mask and further segmented into separated bony structures through a connected component routine. For each component, the center of mass was calculated based on the position of pixels and the amplitude of the ultrasound or photoacoustic image, which was normalized over the maximum amplitude of each component.

#### 5) Landmark Registration:

Pre-operative and post-operative CT volumes (512 × 512 × 192 samples) of the human cadaver were acquired with an O-arm O2 (Medtronic, Minnesota, USA) using 140 kV-peak and 0.78 × 0.78 × 0.83 mm^3^ voxel resolution. The CT volumes were optimized for bone visualization by adjusting the window level to 2000 Hounsfield units (HU) and the window width to 2000 HU. Centers of mass calculated from both ultrasound and photoacoustic images were used as fiducial markers for landmark registration, which was conducted with 3D Slicer [48]. The corresponding fiducial markers in the CT volume were manually placed to match bony contours in the registered CT slices to those in the ultrasound images. The registered CT volume was displayed in X-Z and Y-Z views, where X, Y, and Z represent the lateral, elevation, and axial dimensions of the ultrasound probe.

#### 6) Cancellous Vs. Cortical Bone Differentiation:

Photoacoustic imaging was used to differentiate signals originating from cortical and cancellous bone. Photoacoustic signals from cancellous bone were acquired when the tip of the optical fiber was either touching cancellous bone after being placed within a correctly created pedicle hole or touching the cortical bone surrounding walls of the pedicle after creating an intentional medial or lateral breach. Medial and lateral breaches in the cortical bone were confirmed with the CT volume described in Section II-C5.

SNR was calculated to determine which signals would be included in the analysis of bone differentiation, using the equation:

(8) $$\text{SNR}=\frac{\mu_{t}}{\sigma_{b}},$$

where *μ*_*t*_ and *σ*_*b*_ are the mean and standard deviation of signals within photoacoustic target and background regions of interest (ROIs), respectively, prior to log-compression. To identify appropriate target ROIs, LW-SLSC images were used to estimate the center of the photoacoustic targets (which was challenging with DAS photoacoustic images because of the diffuse patterns observed in some cases [21]). Then, a 10 mm × 10 mm ROI was centered on the photoacoustic target and a background ROI was placed 25 mm above the center of the target.

As demonstrated in the Appendix, photoacoustic acquisitions that yielded a SNR value of 3 or less were considered as out-of-plane signals to be discarded from additional analysis. We reasoned that signals with SNR > 3 were more likely to be associated with a photoacoustic signal from the fiber tip, while SNR values below this threshold produced images that mostly contained noise. These noisy images were suspected to result from signal sources located outside of the imaging plane.

After removing the out-of-plane signal cases, 6 cases of cancellous bone and 5 cases of cortical bone were analyzed. For each case, DAS, SLSC, and LW-SLSC photoacoustic images were processed with the same parameters as described in Section II-C3. Then, contours of −6 dB were computed around a 10 mm × 10 mm ROI that was centered on the photoacoustic target. This process was repeated for 10 acquired frames from each cortical and cancellous bone case. Finally, a t-test was used to evaluate the statistical significance (p < 0.01) of the difference in areas generated from the contours measured when the optical fiber was touching either cancellous or cortical bone. This statistical analysis was repeated for each beamformer.

### D. Image Quality Assessments and Data Representation

The generalized contrast-to-noise ratio (gCNR) [49], [50] was used to assess the separability of bone structures and surrounding soft tissue in ultrasound images, defined as:

(9) $$\text{gCNR}=1-\sum_{x=0}^{1}\underset{x}{\min}\left\{p_{i}(x),p_{o}(x)\right\},$$

where *p*_*i*_ and *p*_*o*_ are the probability density functions of signal amplitudes within regions of interest (ROIs) inside and outside of the lamina, respectively. The probability density functions were calculated from histograms computed with 256 bins. Similarly, the contrast-to-noise ratio (CNR) was measured and compared, defined as:

(10) $$\text{CNR}=\frac{\left|S_{i}-S_{o}\right|}{\sqrt{\sigma_{i}^{2}+\sigma_{o}^{2}}},$$

where *S*_*i*_ and *σ*_*i*_ are the mean and standard deviation, respectively, within a ROI inside of the target prior to log-compression and *S*_*o*_ and *σ*_*o*_ are the mean and standard deviation, respectively, of a ROI outside of the target prior to log-compression.

Results from measurements of the thickness of segmented lines (Section II-B) and from areas of photoacoustic signal originating from cancellous and cortical bone (Section II-C6) are both presented as box-and-whiskers plots in Section III. In these plots, the horizontal lines represent the median, the upper and lower edges of each box represents the upper and lower quartiles of each data set, the top and bottom lines extending from the boxes indicate the maximum and minimum of each data set, and the crosses indicate outliers (defined as any value larger than 1.5 times the interquartile range).

## III. Results

### A. Bony Segmentation of an Ex Vivo Caprine Vertebra

**Fig. 3.**
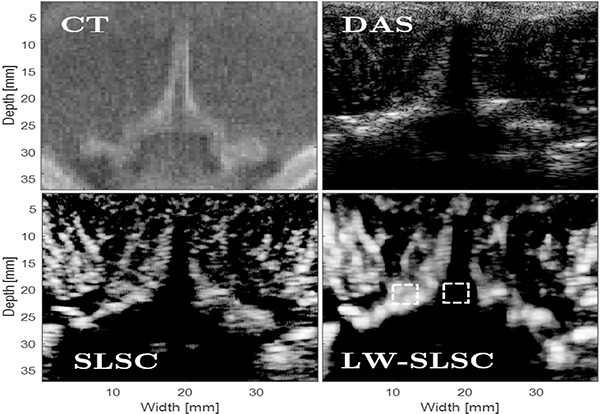
Examples of reconstructed CT, DAS, SLSC (M = 9) and LW-SLSC (*N*_*L*_ = 28, *α* = 0.1) images of the caprine sample (not registered). Regions selected for gCNR measurements are denoted by the dashed boxes.

Fig. 3 shows examples of CT, DAS, SLSC and LW-SLSC images of the *ex vivo* caprine thoracic section. The SLSC and LW-SLSC images were computed with parameters that maximized gCNR, yielding values of 0.98 and 0.99, respectively, for the selected regions of interest. The gCNR of the DAS image was 0.67. In addion to improving gCNR, SLSC and LW-SLSC imaging improved the boundary between soft tissue and the spinous, lamina, and transverse processes of the vertebra, when compared to DAS imaging. CNR was also enhanced in the SLSC and LW-SLSC images (2.13 and 4.59, respectively), when compared to that of the DAS image, which was 0.55.

**Fig. 4.**
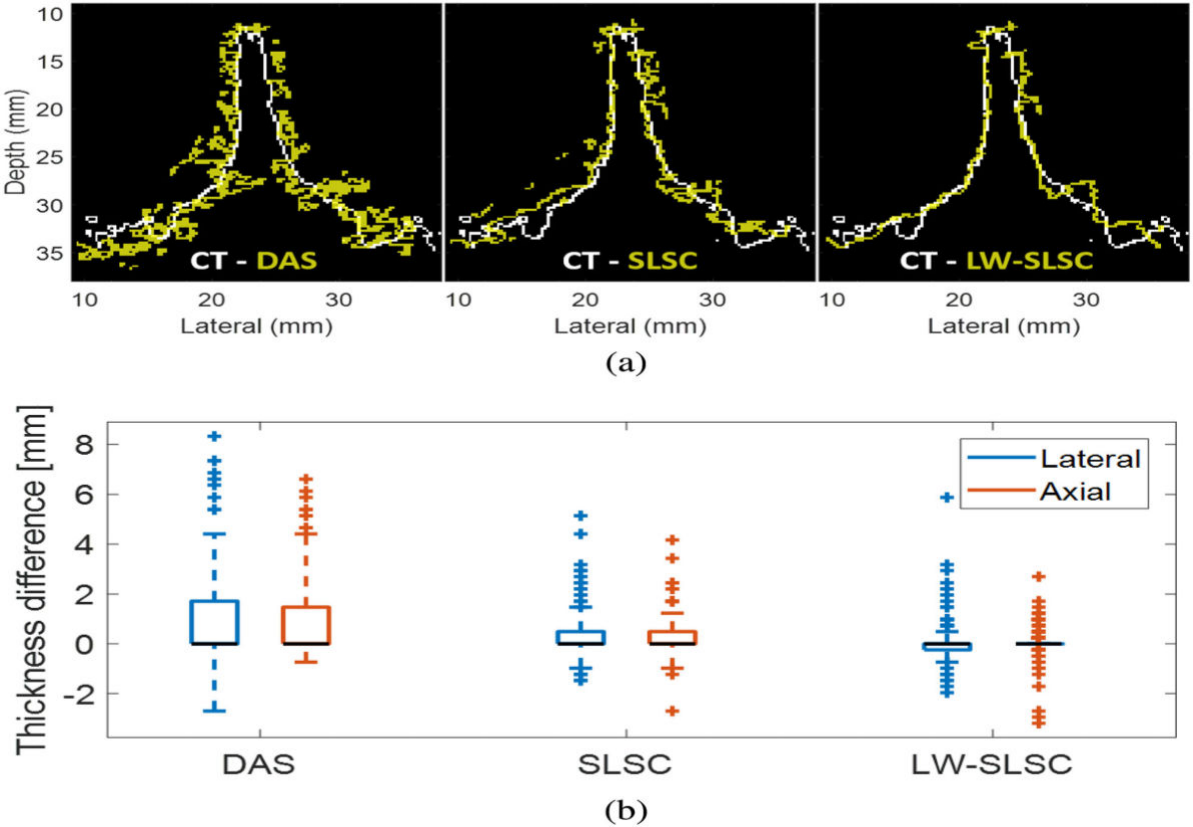
(a) Registered US-CT bone boundaries after applying threshold segmentation to images of the ex vivo caprine vertebra. The ultrasound images were beamformed using DAS (left) SLSC (middle) and LW-SLSC (right). (b) Differences in the integrated thickness of the segmented bone boundary in lateral and axial dimensions, when comparing CT results to DAS, SLSC, and LW-SLSC results. Each boxplot shows the median (horizonal black line), interquartile range, maximum and minimum values of the differences in the integrated thickness.

Fig. 4(a) shows the registration of vertebral boundaries segmented from CT and ultrasound images. While the segmented boundaries successfully converged in the final ultrasound-to-CT registration, a notable difference was observed with DAS when compared to SLSC and LW-SLSC boundaries. Specifically, a fuzzier segmentation was produced from the DAS image, while the coherence-based methods reduced outliers and produced finer contours. An additional reduction of pixel outliers is observed for the LW-SLSC image result which more closely follows the CT contour when compared to SLSC image result.

Fig. 4(b) shows the corresponding thickness difference for the lateral and axial dimension of the segmented boundaries. To quantitatively compare the thickness of the segmented boundaries, the integration of the segmented regions was calculated in the axial and lateral dimensions for each boundary. The differences between these integrated segmentation thicknesses at each lateral or axial position was computed to compare the obtained CT boundary with each of the ultrasound boundaries. The overall thickness of the CT contour in each dimension (axial: 1.84 mm, lateral: 1.79 mm) was closer to that obtained from the LW-SLSC image (axial: 2.09 mm, lateral: 2.03 mm) than that obtained from the SLSC image (axial: 2.98 mm, lateral: 2.89 mm) or DAS image (axial: 5.86 mm, lateral: 5.67 mm).

### B. Vertebral Imaging of a Human Cadaver

**Fig. 5.**
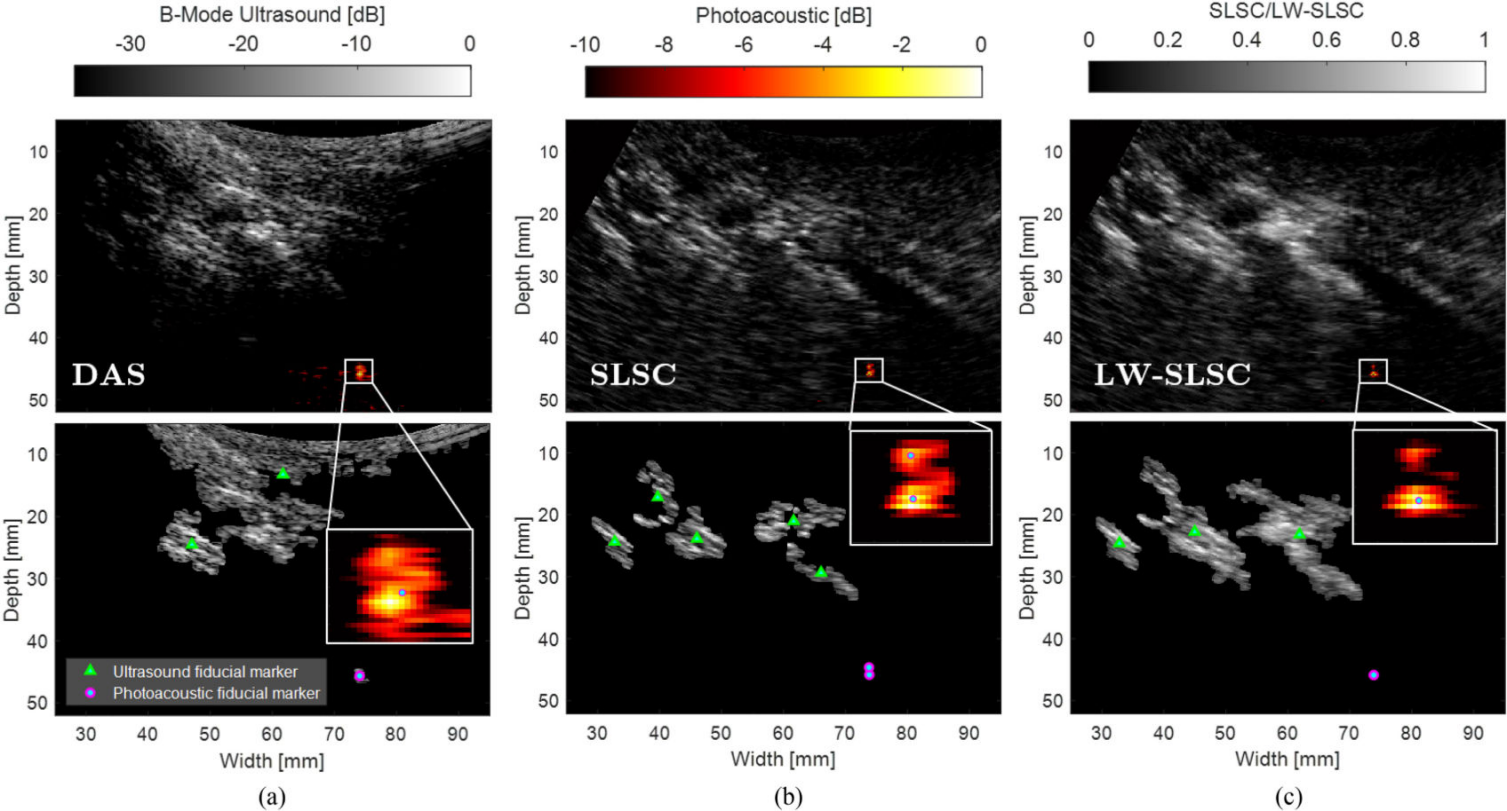
Examples of photoacoustic images overlaid on ultrasound images from an oblique view of L3-L5 vertebrae reconstructed with (a) DAS, (b) SLSC and (c) LW-SLSC ultrasound and photoacoustic beamforming. Top row: beamformed images. Bottom row: segmented masks. The triangles and circles represent the center of mass of isolated components from ultrasound and photoacoustic images, respectively, which are later combined and used as landmarks for CT registration. The insets show magnified views of the photoacoustic signal originating from the fiber tip.

Fig. 5 shows examples of ultrasound and photoacoustic images from a lumbar vertebra inside an intact human cadaver. The top row shows the photoacoustic images overlaid on ultrasound images created with DAS, SLSC, and LW-SLSC beamforming. The discrimination of bone structures in the ultrasound images was determined using the ROIs shown in the Appendix (not shown in Fig. 5 to facilitate the comparison between matched images). SLSC and LW-SLSC beamforming produced average gCNR values of 0.98 and 0.88, respectively, which were both higher than the average 0.77 gCNR calculated from corresponding DAS images. The enhancement of bone visualization is additionally confirmed with the average CNR values, which measured 1.17, 1.75, and 2.68 in DAS, SLSC, and LW-SLSC images, respectively. A summary of the individual gCNR and CNR measurements is presented in Table I.

The photoacoustic signals in Fig. 5 are shown registered to the ultrasound images, with a magnified view shown as a figure inset. These photoacoustic signals arise from the tip of the optical fiber that was inserted into the prepared pedicle hole. Coherence-based images were qualitatively observed to produce more focused photoacoustic signals when compared to DAS photoacoustic images, which is expected to enhance the estimation accuracy of the fiber tip location. Quantitatively, the distance between the center of mass and the brightest pixel of each photoacoustic image created with DAS, SLSC, and LW-SLSC beamforming was 0.26 mm, 0.21 mm, and 0.18 mm, respectively, where a shorter distance represents a more compact and less diffuse photoacoustic signal.

The bottom row of Fig. 5 shows the segmented ultrasound and photoacoustic masks for the three beamformers. The green triangles and magenta circles represents the center of mass of the isolated components from ultrasound and photoacoustic masks, respectively. The segmented masks from the DAS ultrasound image includes undesirable soft tissue and a single bony structure, while coherence methods identify at least 3 bony structures. Similarly, SLSC images created with greater *M* values have an increased number of outliers (i.e., pixels with coherence values that differ significantly from their surroundings and from their values at other lags [41]) and decreased SNR and CNR [39], which caused some otherwise continuous bony structures to appear disconnected, affecting the estimation of center of mass and resulting in redundant landmarks. This effect is mitigated with LW-SLSC.

**Fig. 6.**
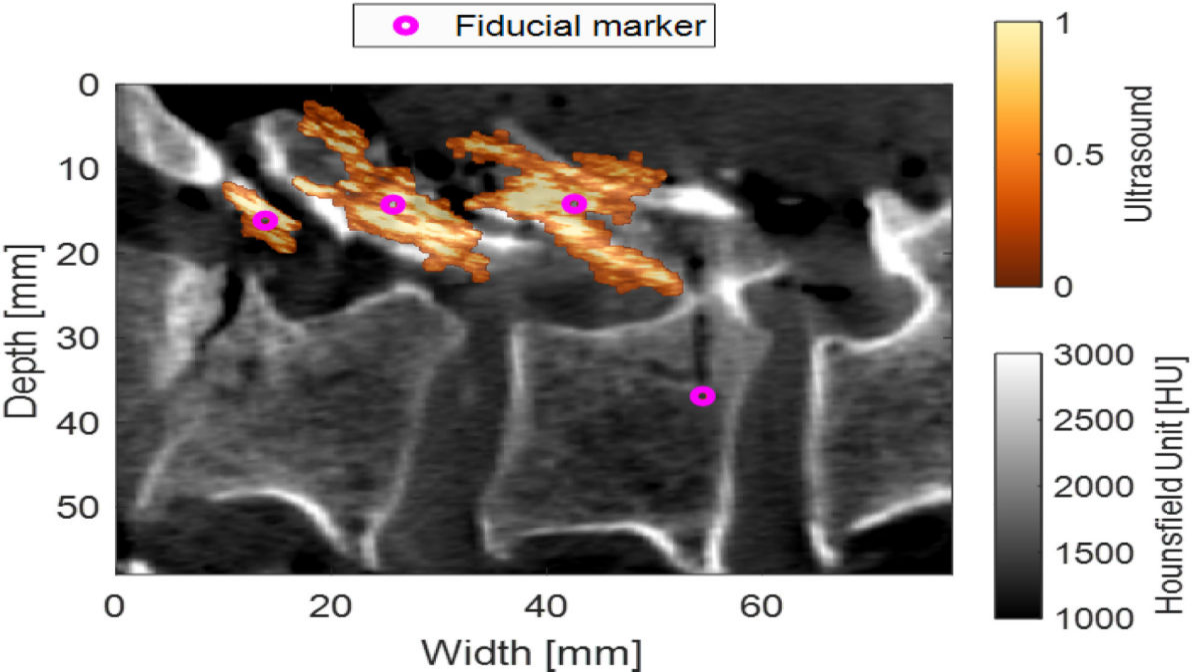
Co-registered ultrasound (color) and CT (grayscale) images using ultrasound and photoacoustic landmarks (magenta) from segmented LW-SLSC images.

Fig. 6 shows the registration of the post operative CT volume with the landmarks obtained from the segmented ultrasound and photoacoustic LW-SLSC images. The shape of the segmented LW-SLSC ultrasound image closely resembles that of the lamina of the L3, L4 and L5 vertebrae in the CT image. Similarly, the fiducial markers for the photoacoustic signals originating from the optical fiber is visualized near the end of the pedicle hole.

**Fig. 7.**
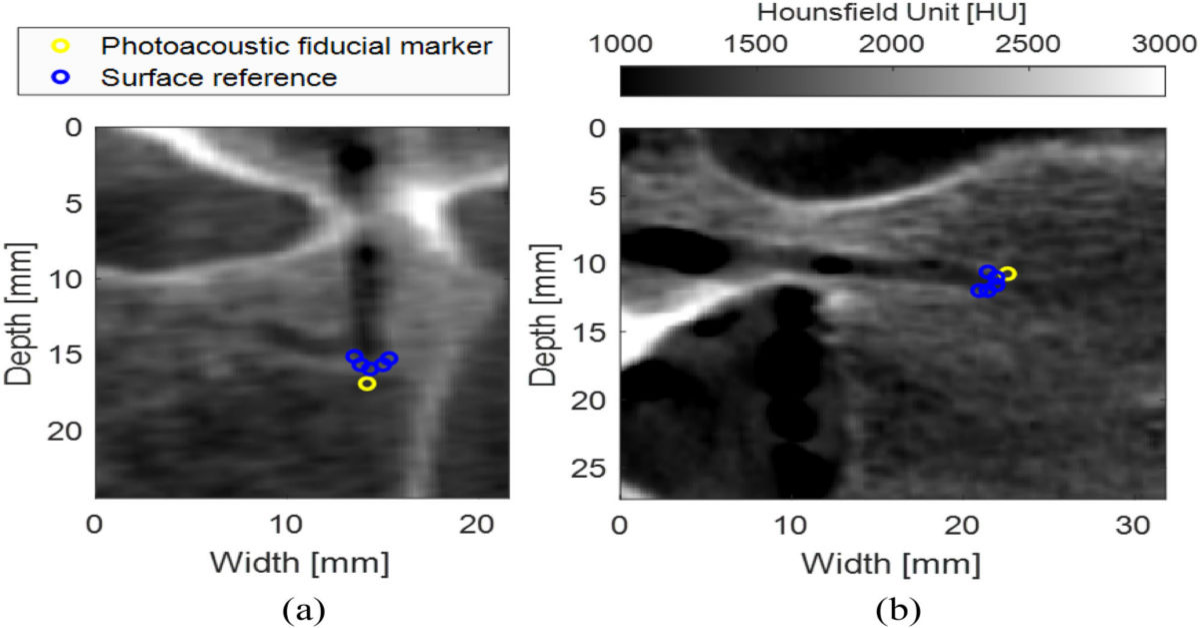
(a) X-Z and (b) Y-Z planes of the CT volume registered to the ultrasound and photoacoustic images. The yellow marker represents the centroid of the photoacoustic signal reconstructed with the LW-SLSC image, which was used as a fiducial marker for landmark registration. The blue markers show the outline of the pedicle hole.

Fig. 7 shows the X-Z and Y-Z views of the registered CT volume and the fiber tip fiducial marker segmented from the LW-SLSC photoacoustic image. To assess the proximity of the registered fiducial marker to the bottom of the pedicle hole, five manual markers were selected around the border of the pedicle hole for each X-Z (Fig. 7(a)) and Y-Z view (Fig. 7(b)). The position of the manual markers represents the potential positions of the optical fiber tip when it was inserted in the pedicle hole. Euclidean distances between the fiducial marker and each of the manual markers are reported in Table II. The minimum distances are shown in bold, indicating the marker associated with the location of the bone surface that the tip of the optical fiber was most likely touching when inserted in the pedicle hole.

**Fig. 8.**
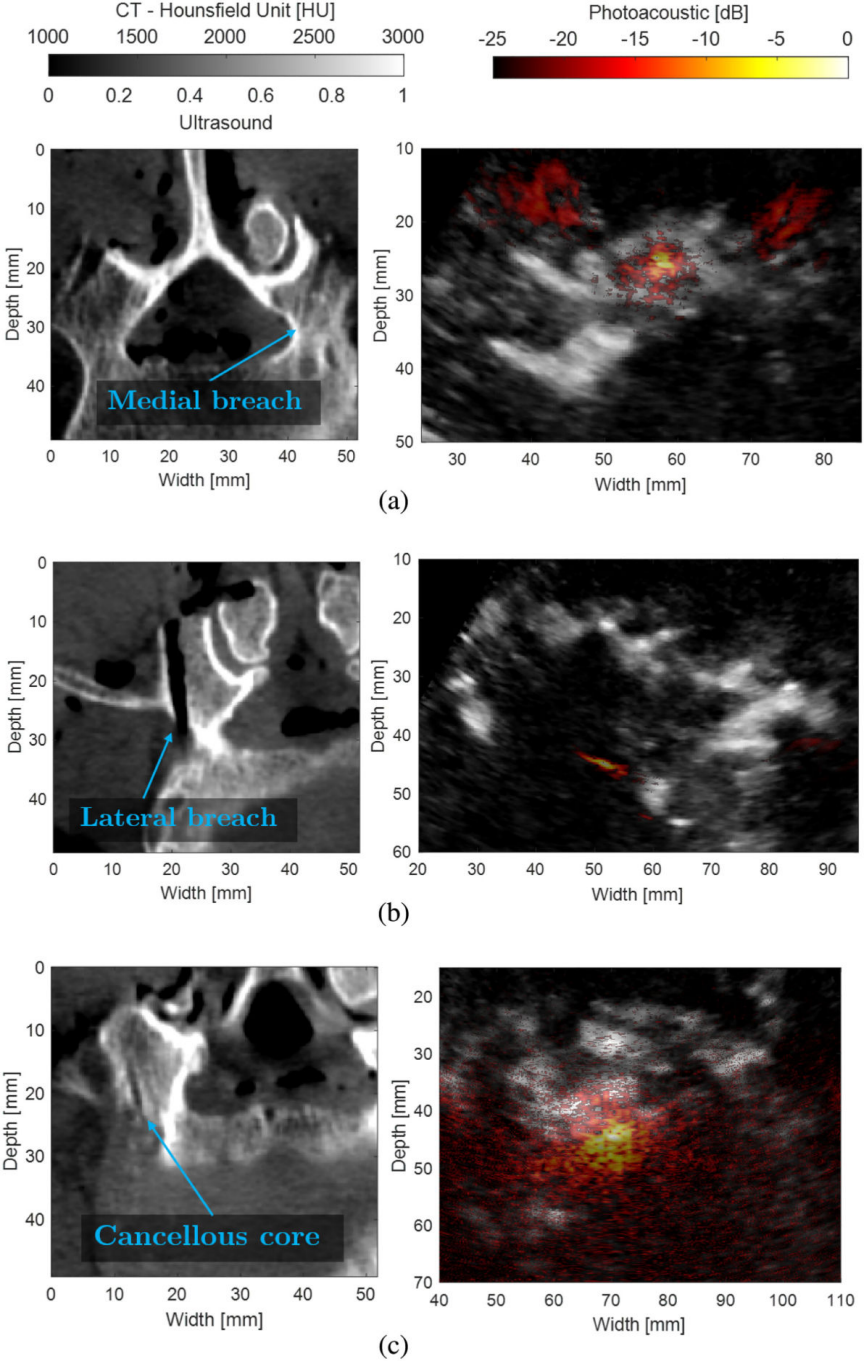
Examples of photoacoustic signals generated when the tip of the optical fiber is touching a (a) medial breach, (b) lateral breach, and (c) cancellous core. Left column: CT axial slice. Right column: LW-SLSC ultrasound image co-registered with DAS photoacoustic image.

Fig. 8 shows examples of co-registered LW-SLSC ultrasound images and DAS photoacoustic images when the tip of the optical fiber was placed in holes corresponding to a medial breach (Fig. 8(a)), a lateral breach (Fig. 8(b)), and the cancellous core of the pedicle (Fig. 8(c)). The corresponding CT slices were chosen to optimize visual confirmation of the fiber placement description, and therefore they are not registered to the photoacoustic and ultrasound images. It was not possible to perform ultrasound-to-CT registration for these figures, because of the absence of clear anatomical landmarks in the ultrasound image of the lumbar vertebrae. Our primary goal was instead to obtain ground truth images while touching the tip of the hole identified by post-operative CT images, without regard to the presence of suitable bony landmarks in the ultrasound images. Axial slices of the CT volume are shown in Fig. 8 in order to clearly visualize the pedicle hole and intentional lateral and medial breaches.

In particular, the medial breach in the CT image of Fig. 8(a) shows the tip of the hole coinciding with high density bone (i.e., the cortical bone) where the tip of the optical fiber was placed. Similarly, the tip of the fiber is in close proximity to the outer cortical wall of the pedicle in Fig. 8(b). In contrast, the tip of the hole in Fig. 8(c) is surrounded by low density bone (i.e., cancellous bone). Qualitatively, DAS photoacoustic images show distinct pattern differences when the optical fiber was touching either cancellous or cortical bone. Specifically, DAS photoacoustic signals from the cancellous core produced signals with greater area coverage than that present with lateral and medial breaches (i.e., fiber touching cortical bone) when images were displayed with the same dynamic range of 25 dB. Because coherence-based methods reduced the appearance of incoherent signals, the area of photoacoustic signals originating from cancellous bone (see Fig. 5) was reduced when compared to the same signals in DAS photoacoustic images, resulting in reduced differentiation between these signal origins with the coherence-based images.

**Fig. 9.**
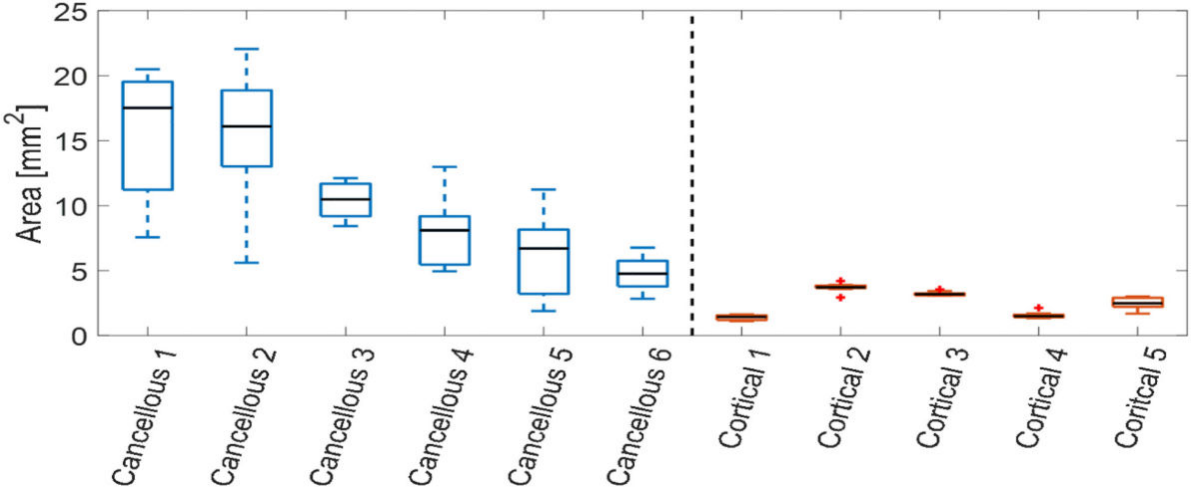
Areas of −6 dB-contours around the center of photoacoustic targets from cortical and cancellous core using DAS beamforming. Each boxplot shows the median, interquartile range, maximum and minimum values of the estimated areas over 10 frames for cancellous (left) and cortical (right) bone.

Fig. 9 shows quantitative comparisons of the differences observed in Fig. 8, as measured by the enclosed area of the −6 dB contours generated from DAS photoacoustic images. These results are grouped by the expected location of the optical fiber tip, touching either cortical or cancellous bone, based on the corresponding CT images. The total mean area measured within the −6 dB-contours was 7.59 mm^2^ greater when touching cancellous bone compared to cortical bone (p < 0.01). In addition, greater standard deviations in these measurements were observed for cancellous bone (5.22 mm^2^) when compared to cortical bone (0.96 mm^2^).

**Fig. 10.**
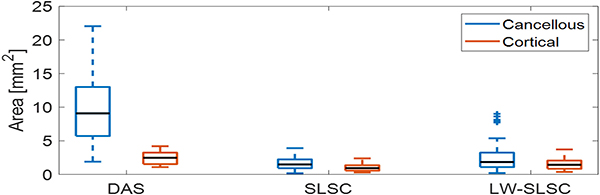
Comparison of −6dB-contours from photoacoustic targets inside cortical and cancellous bone in a human cadaver vertebrae using DAS, SLSC and LW-SLSC beamforming. Each boxplot shows the median, interquartile range, maximum and minimum values of the estimated areas over 60 frames for cancellous and 50 frames for cortical bone.

Fig. 10 compares areas of the −6 dB contours obtained from DAS, SLSC, and LW-SLSC images of the optical fiber touching either cortical or cancellous bone. The mean ± one standard deviation of measurements from DAS images was 10.06 ± 5.22 mm^2^ for cancellous bone and 2.47 ± 0.96 mm^2^ for cortical bone. In comparison, the mean ± one standard deviation of measurements from SLSC images was 1.64 ± 0.88 and 1.06 ± 0.59 mm^2^ for cancellous and cortical bone, respectively. The mean ± one standard deviation of measurements from LW-SLSC images was 2.60 ± 2.25 and 1.51 ± 0.77 mm^2^, for cancellous and cortical bone, respectively. While the three beamformers showed statistically significant differences between the mean of measured areas from cortical and cancellous bone (p < 0.01), DAS images offered the greatest distinction.

## IV. Discussion

We successfully demonstrated that combined ultrasound and photoacoustic imaging has the potential to improve pedicle screw placement during posterior spinal fusion surgeries. Coherence-based beamforming plays an important role in both ultrasound and photoacoustic image formation for this task. Specifically, coherence-based ultrasound imaging improves the visualization of bone structures (Figs. 3 and 5), which enables individual landmarks for each independent bone structure during the registration of ultrasound to CT images (Figs. 5 and 6). As a complement to this information, coherence-based photoacoustic imaging enables localization of fiber tips (Fig. 5).

On the other hand, amplitude-based methods such as DAS photoacoustic imaging of signals inside the lumbar vertebrae allowed differentiation between cortical and cancellous bone. As observed in Fig. 8, DAS photoacoustic images show a diffuse pattern when the optical fiber was inside the pedicle, where its core is composed of cancellous bone. This pattern is understandable, as reflections within the porous, blood-rich structure of the cancellous bone are expected to compromise the alignment of the delayed signals during the beamforming process. In contrast, a well-defined, compact signal was observed for the medial and lateral breaches, which can be explained by the wall surrounding the pedicle being composed by cortical bone, which is more dense than cancellous bone [51] and is expected to produce less signal reflections. Similar signal appearance differences were previously obtained prior to the removal of any bone, presenting photoacoustic imaging as a potential option to find the ideal starting points for pedicle screw insertion [21]. The new contributions of this work demonstrate that these same differences in bone appearance can be used to determine if the pedicle hole is being created with the correct trajectory to avoid impending bone breaches. As out-of-plane signals need to be identified and excluded for successful implementation of this concept, the use of a 2D ultrasound array to identify the out-of-plane photoacoustic signals is a promising alternative to our empirical SNR>3 threshold.

We additionally note that coherence-based beamforming was not sufficient to visualize nor quantify differentiation between cortical and cancellous bone (Fig. 5). These coherence-based beamformers reduced the incoherent signals associated with the cancellous bone, which is a necessary feature of bone differentiation that is emphasized with amplitude-based beamforming methods. However, the added value of coherence-based beamforming is its ability to localize the coherent signal source with more clarity for photoacoustic signal tracking during pedicle hole creation. Thus, we conclude that amplitude- and coherence-based photoacoustic beamformers are synergistically and mutually beneficial for the clinical task of guiding spinal fusion surgeries. Specifically, SLSC and LW-SLSC beamformers have the potential to improve target localization that is otherwise difficult in the presence of noise [37] or diffuse patterns from the cancellous core of the pedicle [21], while DAS beamforming can assist with determining proximity to cortical bone based on the shape of the amplitude-based signal.

In a previous study, a single vertebra with tissue attachments removed was submerged in a water tank [52], and the presence of reverberations required the introduction of some assumptions about fiber tip positions in order to estimate true locations within pre-drilled pedicle holes. However, the human cadaver study presented in this manuscript did not require these additional assumptions. As observed in Figs. 5 and 8, photoacoustic signals from the optical fiber tip did not produce additional artifacts that would otherwise negatively impact tip position estimates (compared with Fig. 2 in [52]). While the previous study differed from the cadaver study by using a custom drill bit that surrounded the optical fiber, we hypothesize that reverberations in [52] were primarily generated by the absence of muscle, nerves, fat, and blood vessels. These additional artifacts were substantially reduced in the human cadaver experiments because of sound attenuation in the surrounding soft tissue, which emphasizes the importance of conducting cadaver studies on the path to clinical translation of this photoacoustic-guided surgery concept, as noted in [53].

Regarding real-time capabilities, DAS and SLSC or LW-SLSC photoacoustic images can be interleaved during surgeries. Previous work describing a real-time GPU implementation of the SLSC beamformer on a research ultrasound system indicates that this is a viable possibility [37]. We demonstrated that photoacoustic SLSC images can be displayed in high-noise-level environments generated with <200*μ*J laser energies at 41 frames per second [37]. Given that LW-SLSC operates on independent kernels ${\hat{R}}_{i}$ as described in Section II-A2, real-time imaging can be similarly achieved by concurrent execution of each ${\hat{R}}_{i}$ in a separate thread inside the GPU. The complexity of the operations per thread is further reduced by pre-computing matrix *B* and *α*^2^*D*^*T*^ *D*, which are defined in Section II-A2. With a GeForce GTX Titan X graphic card, we estimated a computation time of 60 ms based on the number of cores of the GPU (i.e., 3072 cores) and the computation time when executed in MATLAB (i.e., approximately 3 minutes). This estimation does not consider memory transfer and pre-computation times. In addition, we previously developed a deep neural network architecture (i.e., CohereNet) to estimate spatial coherence functions [54], which are foundational to LW-SLSC imaging. This deep learning approach achieved real-time computational processing times and can potentially be adapted to include the additional regularization steps needed for LW-SLSC imaging.

We envision several implementation possibilities to achieve the stated benefits of combined amplitude- and coherence-based ultrasound and photoacoustic images. First, as the fiber tips are ultimately envisioned to be inserted into the hollow core of custom drill bits [21], [36], [52], [55], the observed benefits of coherence-based photoacoustic images can potentially be extended to benefits for tracking the tips of common surgical tools used during spinal fusions surgeries (e.g., drill tips, pedicle probe tips). The feasibility of this concept was demonstrated for drill bits in a previous publication from our group [55]. As observed in Fig. 2 of [55], a stationary optical fiber was connected to the laser source, and the opposite end of the fiber was inserted into a stationary interface. The other end of this stationary interface accommodated a rotating drill bit, which was custom-fabricated with holes on both ends to house a rigidly attached optical fiber that rotated with the drill bit. Both the stationary and rotating optical fibers were air coupled to each other to permit light transmission from the stationary laser to the tip of the rotating drill bit. If attachment to tool tips are not possible, a surgeon may periodically check trajectories by removing the pedicle probe (or any other surgical instrument used to create pedicle holes) and replacing the instrument with an optical fiber, as implemented for the human cadaver study described in this paper.

## V. Conclusion

This paper presents the first known combined ultrasound and photoacoustic image guidance system with software capabilities that are optimized for pedicle cannulation in posterior spinal fusion surgery, demonstrating that both amplitude- and coherence-based beamforming methods are mutually beneficial for this task. Specifically, coherence-based beamforming of ultrasound images improved the visualization of bone for ultrasound-to-CT registration, while coherence-based beamforming of photoacoustic images has the potential to improve target localization and tracking during pedicle hole creation. Amplitude-based photoacoustic beamforming has the potential to provide complementary quantitative information regarding proximity to the cortical bone surrounding the desired pedicle hole trajectory. Overall, this proposed combination of imaging modalities and beamforming methods is promising to assist surgeons with identifying and avoiding impeding bone breaches during spinal fusion surgeries. These new findings are complementary to previous work demonstrating that photoacoustic imaging is useful to determine optimal entry points into the pedicle [21]. Together with these previous findings, we have successfully demonstrated a complete system that has the potential to significantly impact the standard of image guidance methods for spinal fusion surgery.

## Figures and Tables

**Fig. 11.**
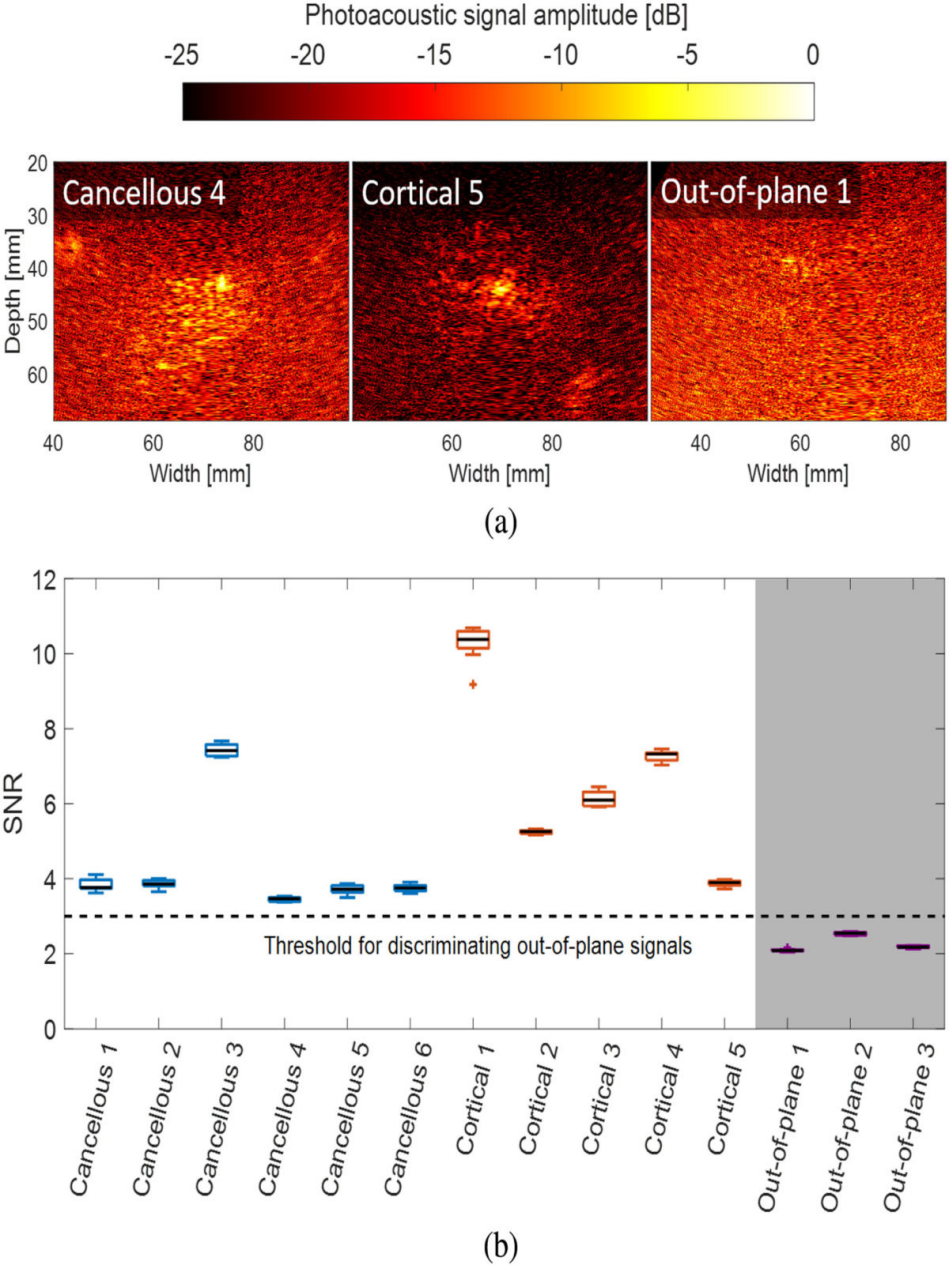
Qualitative and quantitative assessment of photoacoustic images originating from out-of-plane signals. (a) Examples of cancellous, cortical, and out-of-plane DAS photoacoustic images. (b) SNR assessment measured from photoacoustic signals associated with the cancellous core, cortical bone, and characteristic out-of-plane signals. The shaded area represents signals that did not achieve the SNR > 3 threshold and were therefore not included in the area results of Figs. 9 and 10.

**Fig. 12.**
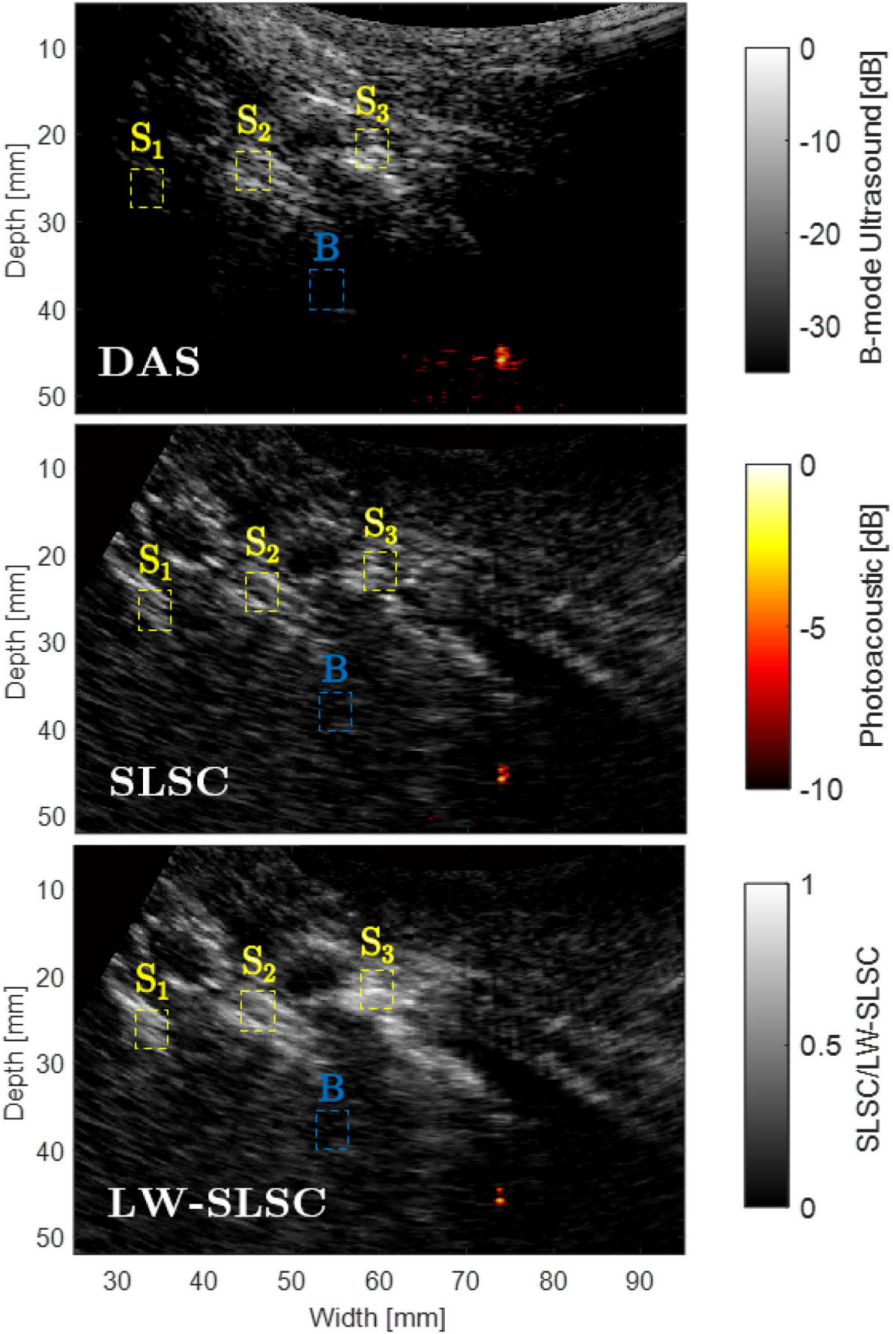
Examples of ultrasound and co-registered photoacoustic images from an oblique sagittal view of L3-L5 vertebrae reconstructed with DAS, SLSC and LW-SLSC. *S*_1_, *S*_2_, *S*_3_, and *B* denote the selected regions for quantitative assessments.

**TABLE I:** Discrimination of Bone Structures in Ultrasound Images of Vertebrae in a Human Cadaver (Determined Using the ROIs Shown in the Appendix)

Metric	Beamformer	*S*_1_	*S*_2_	*S*_3_	Mean

gCNR	DAS	0.34	0.98	0.99	0.77
	SLSC	0.84	0.87	0.93	0.88
	LW-SLSC	**0.98**	**0.99**	**1.00**	**0.98**
CNR	DAS	0.54	2.09	2.10	1.17
	SLSC	1.61	1.64	2.21	1.75
	LW-SLSC	**2.49**	**2.66**	**4.50**	**2.68**

**TABLE II:** Euclidean Distances Between the Fiducial Marker Segmented From the LW-SLSC Photoacoustic Image and Each of the Manual Markers of the Pedicle Hole Obtained From the Registered CT Images in Fig. 7

	X-Z view	Y-Z view

Marker 1	2.02 mm	1.16 mm
Marker 2	1.49 mm	**0.72 mm**
Marker 3	1.91 mm	1.06 mm
Marker 4	1.28 mm	1.69 mm
Marker 5	**0.98 mm**	2.48 mm
Average	1.53 ± 0.39 mm	1.42 ± 0.61 mm

## Appendix

To provide additional justification and rationale for omitted acquisitions, Fig. 11(a) shows examples of DAS photoacoustic images chosen from the lowest SNR cases for the cancellous core, the cortical bone, and signals originating from outside of the imaging plane. The corresponding mean ± one standard deviation SNR were 3.45 ± 0.06, 3.88 ± 0.07, and 2.09 ± 0.03, respectively. In contrast to signals originating from the cancellous or the cortical bone, our observation and experience indicate that of out-of-plane signals are characterized by a high noise level throughout the entire DAS image and a relatively small area coverage around the target identified with LW-SLSC beamforming. SNR measurements were calculated as described in Section II-C6. Fig. 11(b) quantifies the SNR within DAS images from photoacoustic targets originating from the cancellous core, cortical bone, and out-of-plane signals. By empirically defining a threshold SNR of 3, out-of-plane signals were discarded from the area analysis.

To facilitate comparisons between the DAS images and each SLSC and LW-SLSC image, ROIs are not shown in Fig. 5. However, to provide a refrence point, Fig. 12 shows the ROIs used for quantitative assessment of ultrasound image quality reported in Table I.
